# AntagomiR‐29b inhibits vascular and valvular calcification and improves heart function in rats

**DOI:** 10.1111/jcmm.15770

**Published:** 2020-08-26

**Authors:** Ming Fang, Kangyong Liu, Xinming Li, Yudai Wang, Wei Li, Bin Li

**Affiliations:** ^1^ Department of Cardiology Hainan General Hospital Haikou China; ^2^ Department of Cardiology Shanghai University of Medicine & Health Sciences Affiliated Zhoupu Hospital Shanghai China; ^3^ Department of Neurology Shanghai University of Medicine & Health Sciences Affiliated Zhoupu Hospital Shanghai China; ^4^ Department of Radiology Shanghai University of Medicine & Health Sciences Affiliated Zhoupu Hospital Shanghai China

**Keywords:** antagomiR‐29b, miR‐29b, TGF‐β3, vascular calcification

## Abstract

We aimed to investigate the role of the miR‐29b and its effect on TGF‐β3 pathway in vascular and valvular calcification in a rat model of calcific aortic valve diseases (CAVD). A rat model of CAVD was established by administration of warfarin plus vitamin K. The expression levels of miR‐29b, osteogenic markers and other genes were determined by qRT‐PCR, Western blot and/or immunofluorescence and immunohistochemistry. The calcium content and alkaline phosphatase (ALP) activity were measured. The calcium content, ALP activity and osteogenic markers levels in calcified aorta and aortic valve were augmented compared to controls. The expression of miR‐29b, p‐Smad3, and Wnt3 and β‐catenin was significantly up‐regulated, whereas TGF‐β3 was markedly down‐regulated. However, compared with the CAVD model group, the calcium content and ALP activity in rats treated with antagomiR‐29b were significantly decreased, and antagomiR‐29b administration reversed the effects of CAVD model on the expression of miR‐29b and osteogenic markers. Inhibition of miR‐29b in CAVD rats prevented from vascular and valvular calcification and induced TGF‐β3 expression, suggesting that the miR‐29b/TGF‐β3 axis may play a regulatory role in the pathogenesis of vascular and valvular calcification and could play a significant role in the treatment of CAVD and other cardiovascular diseases.

## 1 INTRODUCTION

Calcification of the aortic arch is an independent risk factor for coronary artery disease and increases mortality and morbidity associated with cardiovascular disease such as calcified aortic stenosis and calcific aortic valve disease (CAVD). CAVD is a common condition of cardiovascular diseases in developed countries, affecting up to 3% of people over the age of 65.^1^ The prevalence of CAVD is closely related to risk factors such as age, dyslipidaemia, smoking, diabetes and hypertension.^2^, ^3^ Scientists have demonstrated that vascular calcification (VC) is a complex biological process involving the mineralization and transdifferentiation of vascular smooth muscle cells (VSMCs) by chondrogenic and osteoblastogenic pathways.^4^, ^5^ Calcification of the aortic valve leads to aortic stiffness and systolic hypertension, which constitute an important risk factor of mortality due to cardiovascular diseases.^6^, ^7^ CAVD can be in the asymptomatic phase for a long time and may persist for several years before symptoms appear.^8^ Previous studies have shown that CAVD can be diagnosed at the early stage by Doppler echocardiography and cardiac auscultation,^9^ providing a time window for early treatment. Nonetheless, effective drug therapies have not yet been developed to control the pathogenesis and progression of CAVD and calcification‐related heart conditions. A significant number of previous studies have elucidated the mechanisms associated with the pathogenesis of calcification of the vessels, but much remains to be done for a complete elucidation of the precise mechanisms contributing to the cardiovascular diseases induced by valvular and aortic calcification. This necessitates in‐depth molecular studies.

MicroRNAs (miRNAs) are small non‐coding RNAs (~22 nucleotides) that lead to silencing of genetic information by annealing inexactly to complementary sequences in the 3′‐untranslated regions (3′‐UTR) of the target mRNA causing mRNA destabilization and/or translational inhibition.^10^ Recent advances have identified miRNAs as key regulators of cancer, and they also play an integral role in the pathogenesis of cardiovascular calcification.^4^, ^11^, ^12^ Previous studies have shown that the expression of miR‐29b is decreased in cholecalciferol‐induced rat calcified arteries.^13^ Moreover, miR‐29b‐3p overexpression significantly inhibits arterial calcification through regulating the expression of matrix metalloproteinases‐2 (MMP2) in vivo and in vitro.^13^ In the study of Du et al,^14^ miR‐29 targets the cartilage oligomeric matrix protein‐degrading metalloproteinase, a disintegrin and metalloproteinase with thrombospondin motifs‐7, to inhibit vascular calcification. Additionally, in our recent study, we have identified miR‐29b as an endogenous positive regulator of human aortic valve interstitial cells (VICs) calcification that functions through repressing TGF‐β3 expression in vitro.^15^ However, whether miR‐29b could target TGF‐β3 to attenuate CD and cardiovascular diseases such as CAVD in vivo has yet to be explored.

In this study, we investigated the role of the miR‐29b/TGF‐β3 axis in CAVD in vivo. We found that miR‐29b was significantly induced in CAVD and inhibition of miR‐29b was followed by decreased expression of osteoblastic differentiation and calcification markers and attenuated vascular and valvular calcification through derepressing the TGF‐β3 signalling pathway.

## 2 MATERIALS AND METHODS

### 2.1 Chemicals

Primary antibodies include VEGF (Cat# sc‐80436, RRID:AB_1131207, Santa Cruz Biotechnology), OPN (Cat# sc‐10593, RRID:AB_2270967, Santa Cruz Biotechnology), ALP (Cat# sc‐365765, RRID:AB_10842161, Santa Cruz Biotechnology), OCN (Cat# ab13418, Abcam), Runx2 (Cat# MBS186093, RRID:AB_10888180, MyBioSource), TGF‐β3 (Cat# ab90264, RRID:AB_2050388, Abcam), Smad3 (Cat#NBP1‐88732, RRID:AB_11034477, Novus), Wnt3 (Cat# ab32249, Abcam), β‐catenin (Cat# 26170, RRID:AB_2629235, NewEast Biosciences) and GAPDH (Cat# JM‐3777‐100, RRID:AB_843142, MBL International). LNA anti–miR‐29b oligonucleotides (antagomiR‐29b) and control LNA–scrambled oligonucleotides were custom generated and provided by GenePharma. As a negative control (NC), 10 point mutations were made in the miR‐29b mature sequence generating an RNA sequence that is not expressed in the rat genome.

### 2.2 Rat model of CAVD and experimental groups

A total of 80 male Sprague‐Dawley (SD) rats (8 weeks old, weighing 250‐280 g), purchased from Vital River Laboratory Animal Technology Co. Ltd, were housed at 22 ± 2°C and 40%‐60% humidity with light/dark cycle of 12‐hours. The rats had free access to food and water. The rat model of CAVD was established as described previously with minor modification.^16^ In brief, rats were randomly assigned to control group (n = 20) and CAVD group (n = 60). Rats in CAVD group were treated with warfarin (20 mg/kg/d in drinking water) and subcutaneous injection of vitamin K (15 mg/kg/d to prevent bleeding) on days 1, 3, 5, 7, 14, 21 and 28. Dosages were adjusted every second day. After establishment of CAVD model, the rats in the CAVD model group were arbitrarily distributed in three groups: CAVD group (n = 20), antagomiR‐29b group (n = 20) and antagomiR control (NC) group (n = 20). Animal studies were performed in accordance with the Declaration of Helsinki and approved by the Institutional Animal Care and Use Committee of Hainan General Hospital and conformed to the Guide for the Care and Use of Laboratory Animals.

### 2.3 In vivo administration of antagomiR‐29b

For treatment with antagomiR‐29b, after confirming CAVD in rats, 100 µL of antagomiR‐29b or NC (which were diluted in PBS at 2 mg/mL) was injected to rats in these groups whereas those in the control group and the CAVD group were injected with equivalent volume of PBS three times per week by tail vein injection for 4 weeks.

### 2.4 Measurement of heart of rats

The rats were weighed and placed in an airtight box with 4% pentobarbital (30 mg/kg) for anaesthesia. The chest of the rats was depilated and fixed on the test plate followed by coating an appropriate amount of ultrasonic coupling agent. Subsequently, echocardiography was performed using the Vevo 2100 (VisualSonic). The depth of the image was adjusted to about 2 cm, and the frequency was 21 MHZ. Continuous left ventricular end‐systolic diameter (LVDs), left ventricular end‐diastolic diameter (LVDd), left ventricular ejection fraction (EF%) and fractional shortening (FS%) were measured using M‐mode echocardiography. A normal electrocardiogram (ECG) was recorded following subcutaneous placement of electrodes and connection to an electrocardiograph (BL‐420S; Chengdu Taimeng Technology Co., Ltd.).

### 2.5 Tissue analysis

At day 28, rats were anesthetized with 4% pentobarbital (30 mg/kg) to collect blood plasma. After that, rats were sacrificed by cervical decapitation and portions of aortas and hearts were collected. To collect the aortic roots, rat hearts were fixed with 4% paraformaldehyde in 0.1 mol/L sodium phosphate buffer (pH 7.4) and embedded in paraffin. Then, cross‐sectional slices with a thickness of about 7 µm were prepared. The sections of aortic root located at the proximal of the aortic valve area were selected for histological analysis, so there were only a limited number of slices available before entering the ascending aorta. 5‐mm sections of aorta and aortic root pieces were stained with haematoxylin and eosin (H&E). Data analyses were performed using the ImagePro Plus 6.1 image analysis software (Media Cybernetics).

### 2.6 ALP activity assay

ALP activity in plasma, aorta and aortic roots was detected as previously described.^17^ Briefly, abdominal aortic blood was harvested and mixed with 50 U/mL of heparin. Subsequently, plasma was isolated from the aortic blood by centrifugation at 1409 *g* for 15 minutes at 4°C. Aortic tissue was homogenized in pre‐cooled physiological saline, followed by centrifugation at 7529 *g* for 10 minutes to collect the supernatant. ALP activity of plasma and tissue supernatants was determined using the ALP assay kit (Sigma Chemical Co.), and data analysis was performed as previously described in the Bradford method.^18^

### 2.7 Quantification of calcium content in aortas

The calcium content in aorta and aortic roots was determined using o‐cresolphthalein complexone colorimetric approach. Briefly, the aortas and aortic roots were air‐dried and weighed, followed by addition more than 10 times volumes (w/v) of 100 mL/L formic acid and incubated overnight at 4°C. The supernatant was collected by centrifugation at 3000 rpm for 10 minutes before calcium detection. The reagent and the supernatant were mixed and incubated at 37°C for 5 minutes according to the manufacturer's instruction. Then, the absorbance at a wavelength of 600 nm was measured using a spectrophotometer (Thermo Scientific Biotech) to calculate the calcium deposition content of each group of aortas and aortic roots. Calcium content was determined relative to the dry tissue weight and was expressed in mg/g dry tissue.

### 2.8 Real‐time PCR analysis

Total RNA was extracted from aorta using TRIzol reagent (Invitrogen) and subsequently reverse transcribed into cDNA. Real‐time PCR was performed on the cDNA in a 20 µL reaction system using Mx3000 Multiplex Quantitative PCR System (Stratagene), and the PCR product was assessed using Eva Green fluorescence (Invitrogen). GAPDH was selected as the internal reference gene for mRNA levels whereas U6 was used as the internal reference for miR‐29b levels. The primer sequences are shown in Table 1. The ΔΔ*C*_t_ method was used for computing the relative mRNA expression levels.

**Table 1:** The primer sequences for qRT‐PCR

Primer	5′‐3′
GAPDH‐F	AGGTCGGTGTGAACGGATTTG
GAPDH‐R	TGTAGACCATGTAGTTGAGGTCA
U6‐F	GGTCGGGCAGGAAAGAGGGC
U6‐R	GCTAATCTTCTCTGTATCGTTCC
miR‐29b‐F	TCTGACTTCTCCTGCCTTTACC
miR‐29b‐R	AGACCTGACTGCCATTTGTGAT
ALP‐F	TCCGTGGGCATTGTGACTAC
ALP‐R	TGGTGGCATCTCGTTATCCG
OCN‐F	GCTACCTTGGAGCCTCAGTC
OCN‐R	GGCGGTCTTCAAGCCATACT
Runx2‐F	CATGGCCGGGAATGATGAGA
Runx2‐R	TGAAACTCTTGCCTCGTCCG
OPN‐F	CTGAGGAAACTGATGACAAC
OPN‐R	GCTTTCATTGGACTTACTTGG
TGF‐β3‐F	ATGACCCACGTCCCCTATCA
TGF‐β3‐R	ACTCAGACTCCGAGGTCTCC
Smad3‐F	TGCAGCCGTGGAACTTACAA
Smad3‐R	GACCTCCCCTCCGATGTAGT
Wnt3‐F	ACAACAATGAAGCAGGCCGA
Wnt3‐R	ACTCACGGTGTTTCTCCACC
β‐catenin‐F	CCCAGTCCTTCACGCAAGAG
β‐catenin‐R	CATCTAGCGTCTCAGGGAACA

### 2.9 Western blot analysis

The aortic tissues were washed 3 times with PBS. The total protein was extracted by RIPA lysate (Sigma‐Aldrich). The protein concentration was measured by BCA method. About 40 µg of protein was separated by SDS‐PAGE electrophoresis, and the protein was transferred onto the PVDF membrane (Millipore) by wet transfer method. Subsequently, the membrane was blocked with 5% skim milk at room temperature for 45 minutes followed by the addition with primary antibodies (anti‐ALP, 1:1000; anti‐OCN, 1:1000; anti‐OPN, 1:1000; anti‐Runx2, 1:1000; anti‐TGF‐β3, 1:1000; anti‐Smad3, 1:1000; anti‐Wnt3, 1:1000; anti‐β‐catenin, 1:1000; anti‐GAPDH, 1:1000) and incubation at 4°C overnight. The membrane was washed 3 times with TBST and incubated with the horseradish peroxidase‐conjugated goat anti‐rat immunoglobulin G secondary antibody (Cat# PA1‐28636, RRID: AB_10983589, 1:5000, Thermo Fisher Scientific) for 40 minutes at room temperature. The bands were revealed by the Pierce™ ECL Western Blotting Substrate (Pierce) in accordance with the protocol provided by the manufacturer. Densitometrical analysis was performed using Image J software (version 1.41o, Java 1.6.0_10, Wayen Rasband, US National Institutes of Health), and the relative content of the target protein was expressed by the grey ratio of the target protein/GAPDH.

### 2.10 Immunohistochemical staining

After dewaxing in three toluene baths of 3 minutes each, the sections were rehydrated by passage through decreasing gradients of alcohol baths. After that, the sections were subjected to antigen unmasking by heat in a citrate buffer at pH 6 for 10 minutes. Then, 0.1% sodium azide (Sigma‐Aldrich) was used to inhibit endogenous peroxidases in the presence of 0.3% hydrogen peroxide for 30 minutes. After washing with PBS (Invitrogen), 10% BSA (Dako) was added for a period of 30 minutes. Then, the sections were incubated with anti‐OPN polyclonal primary antibodies (MAb1433, 1:1000, R&D Systems). Isotype antibodies were used as negative controls. After rinsing, a biotinylated rabbit anti‐rat secondary antibody (Dako) was added for 30 minutes; then, a biotinylated streptavidin‐peroxidase complex (Dako) is applied for 30 minutes. Diaminobenzidine tetrahydrochloride (DAB) (Dako) was used for development. Finally, Haematoxylin‐Meyer was used for counterstaining. After image acquisition, ImageJ software was used for quantification.

### 2.11 Immunofluorescence analysis

Frozen aortic sections were fixed and incubated with anti‐VEGF antibodies (rabbit polyclonal antibody, 1:500) and with the fluorescently tagged Alexa fluor‐647 secondary antibody (Alexa fluor‐647 anti‐rat #712‐495‐153, Jackson Immunoresearch). The Zeiss confocal microscope (Zeiss) was used for fluorescence visualization. The 4,6‐diamidino‐2‐phenylindole (DAPI; Molecular Probes)‐containing medium was used for mounting the slides.

### 2.12 Statistical analysis

GraphPad Prism v6.0 (GraphPad Software, Inc) software was used for statistical analysis, and data were expressed as mean ± SD. Comparisons between groups were performed using one‐way or two‐way ANOVA, followed by Newman–Keuls multiple comparison test. *P* < .05 was considered statistically significant, and all experiments were repeated three times.

## 3 RESULTS

### 3.1 Warfarin up‐regulated miR‐29b in CAVD rat model

**FIGURE 1:**
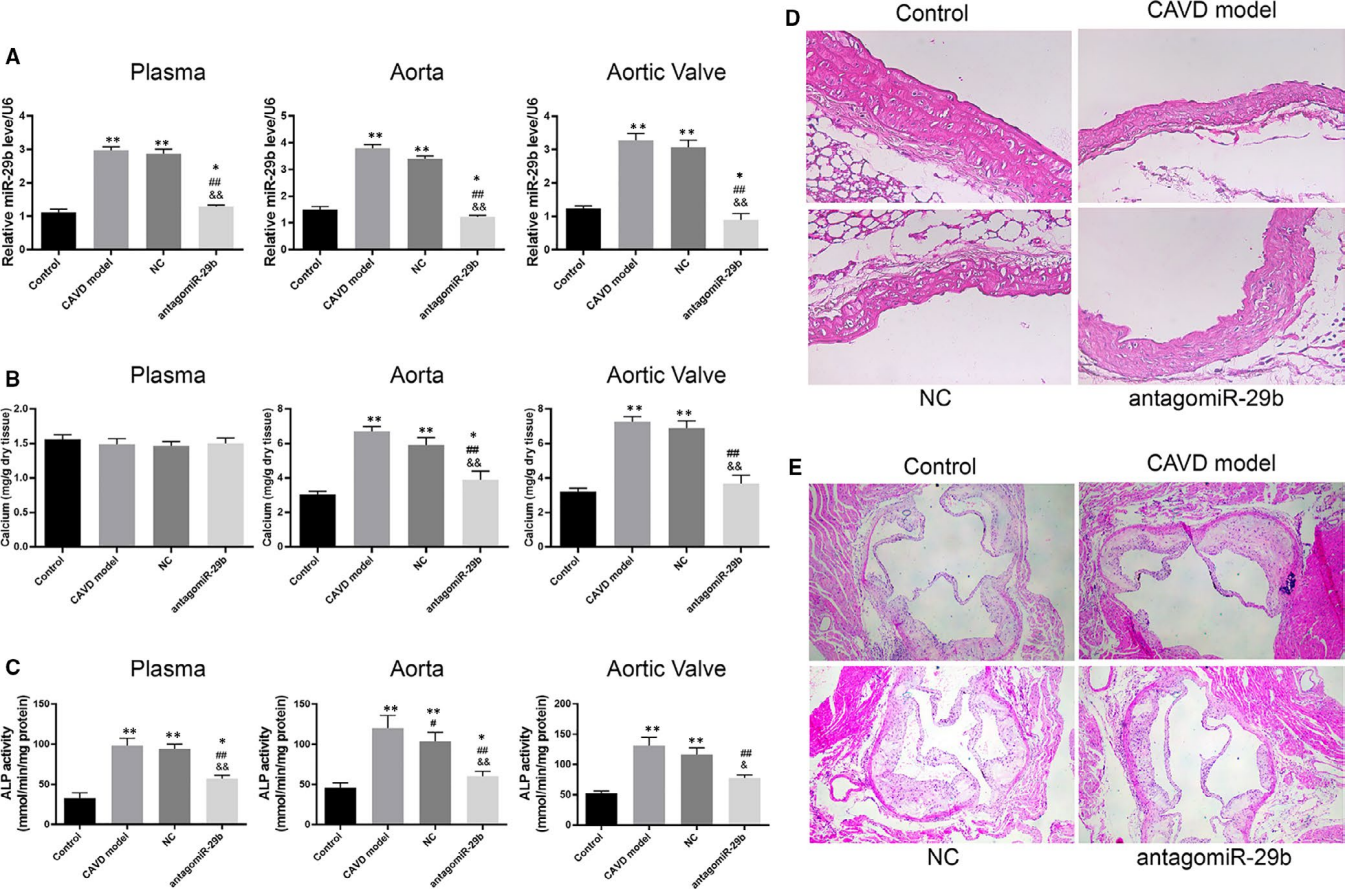
AntagomiR‐29b attenuated the mineralization of vascular tissue in CAVD model. A, Expression of miR‐29b in plasma, aorta and aortic valves in different groups as determined by qRT‐PCR. B, Calcium content in plasma, aorta and aortic valves in different groups. C, ALP activity in plasma, aorta and aortic valves in different groups. D, Representative images of HE staining of aorta sections obtained from different groups. E, Representative images of HE staining of aortic root sections obtained from different groups. The photomicrograph was taken at 100x magnification. The experiments were performed at least 3 times, and data are expressed as means ± SD. *P < .05, **P < .01 vs control group, #*P* < .05, ##*P* < .01 vs CAVD model group, &*P* < .05, &&*P* < .01 vs NC model group

In order to examine the expression of miR‐29b in CAVD rat model, qRT‐PCR experiments were performed in plasma, aorta and aortic valve samples collected from warfarin‐treated and control rats. The results indicated that miR‐29b expression level was significantly increased in the plasma, aorta and aortic valves of CAVD rat model compared to the control rats (Figure 1A).

### 3.2 AntagomiR‐29b attenuated the mineralization of vascular tissue in CAVD model

The increased expression of miR‐29b suggested that this miRNA may play a significant role in CAVD in vivo. Thus, we aimed to investigate its effect on key parameters involved in CAVD. To this achievement, the antagomiR‐29b was injected in CAVD rats. The efficiency of antagomiR‐29b was assessed by qRT‐PCR, and the results showed that the expression levels of miR‐29b in plasma, aortas and aortic valves of CAVD rats were significantly decreased (Figure 1A). Next, we evaluated the effect of antagomiR‐29b on the mineralization of aortas and aortic valves. The measurement of calcium content indicated no significant difference in plasma level of calcium (*P* > .05), but the calcium content in the aorta and aortic valves was significantly higher in the CAVD model group compared to the control group (*P* < .01, Figure 1B). On the contrary, after the treatment with antagomiR‐29b, calcium content was markedly decreased compared to the CAVD model group (*P* < .01, Figure 1B). In addition, compared with control rats, increased ALP activity in plasma, aorta and aortic valves was observed in the CAVD rat model whereas antagomiR‐29b significantly reverted this effect (*P* < .01, Figure 1C). Furthermore, H&E staining revealed disordered elastic fibres in aortas of CAVD group compared with control aortas but this effect was counteracted by antagomiR‐29b (Figure 1D). Similarly, H&E staining of the aortic roots indicated the disorganization of tissue structure in the CAVD rat model compared to the control group whereas counteracting effects were observed in antagomiR‐29b group (Figure 1E) compared to the CAVD model group.

### 3.3 AntagomiR‐29b promoted TGF‐β3 expression and inhibited Wnt3/β‐catenin/Smad3 axis and osteogenic factors in the aorta and aortic valves of CAVD model

**FIGURE 2:**
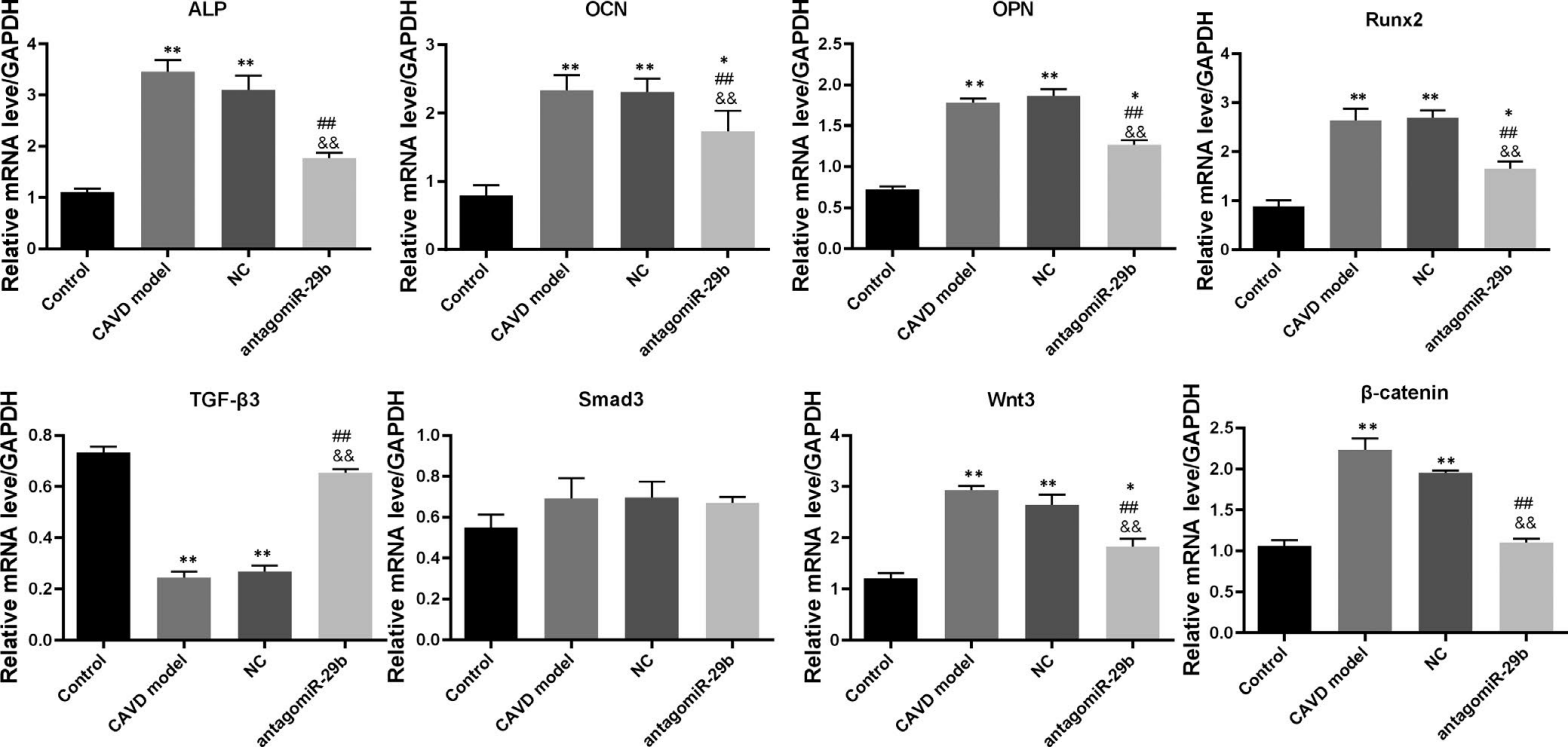
AntagomiR‐29b promoted TGF‐β3 expression and inhibited Wnt3/β‐catenin/Smad3 axis and osteogenic factors in the aorta of CAVD model at transcription level. The mRNA expression levels of different genes were determined by qRT‐PCR. The experiments were performed at least 3 times, and data are expressed as means ± SD. *P < .05, **P < .01 vs control group, ##*P* < .01 vs CAVD model group, &&*P* < .01 vs NC model group

**FIGURE 3:**
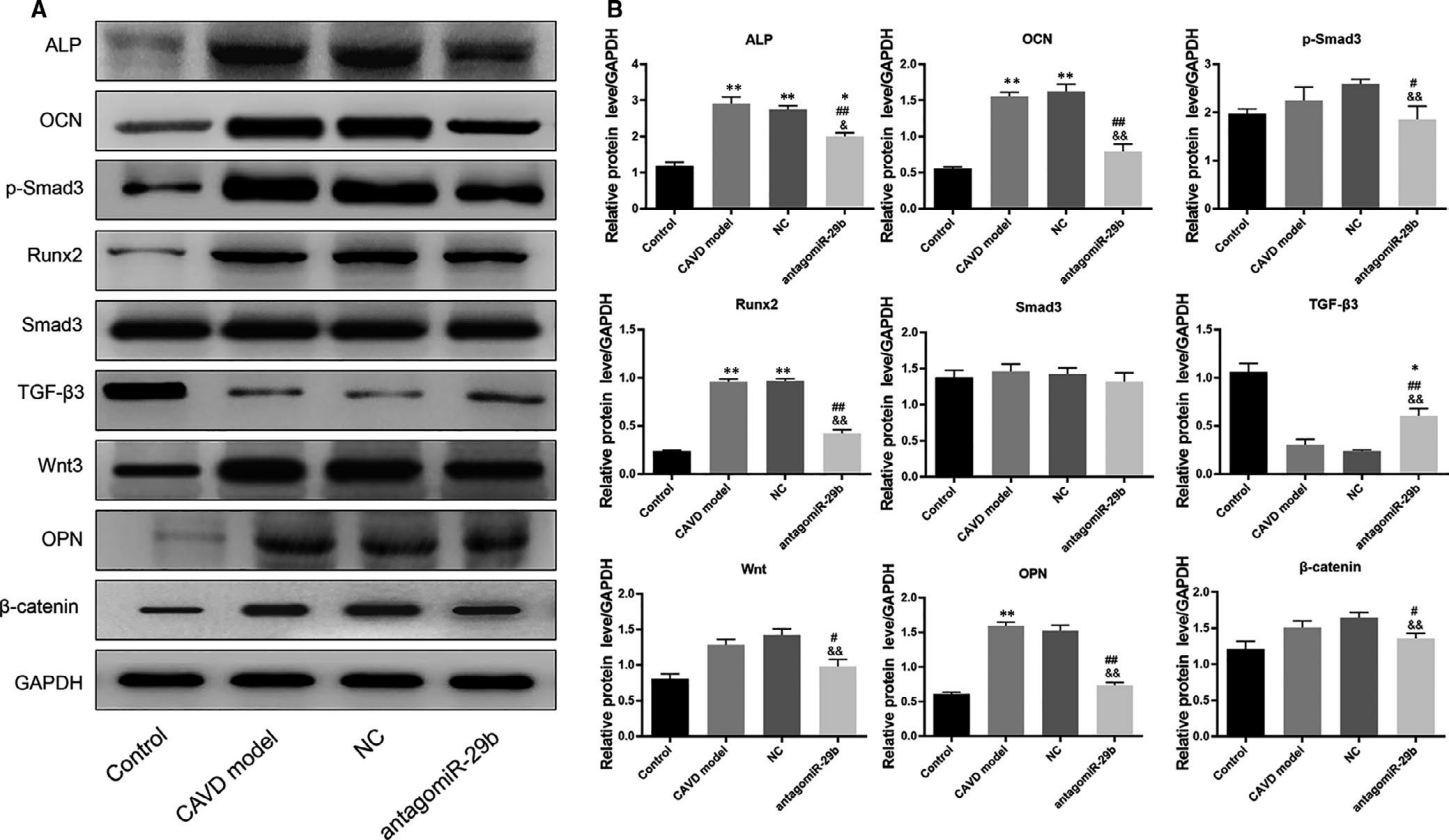
AntagomiR‐29b promoted TGF‐β3 expression and inhibited Wnt3/β‐catenin/Smad3 axis and osteogenic factors in the aorta of CAVD model at protein level. The protein expression levels of different genes were determined by Western blotting. The experiments were performed at least 3 times, and data are expressed as means ± SD. *P < .05, **P < .01 vs control group, #*P* < .05, ##*P* < .01 vs CAVD model group, &*P* < .05, &&*P* < .01 vs NC model group

**FIGURE 4:**
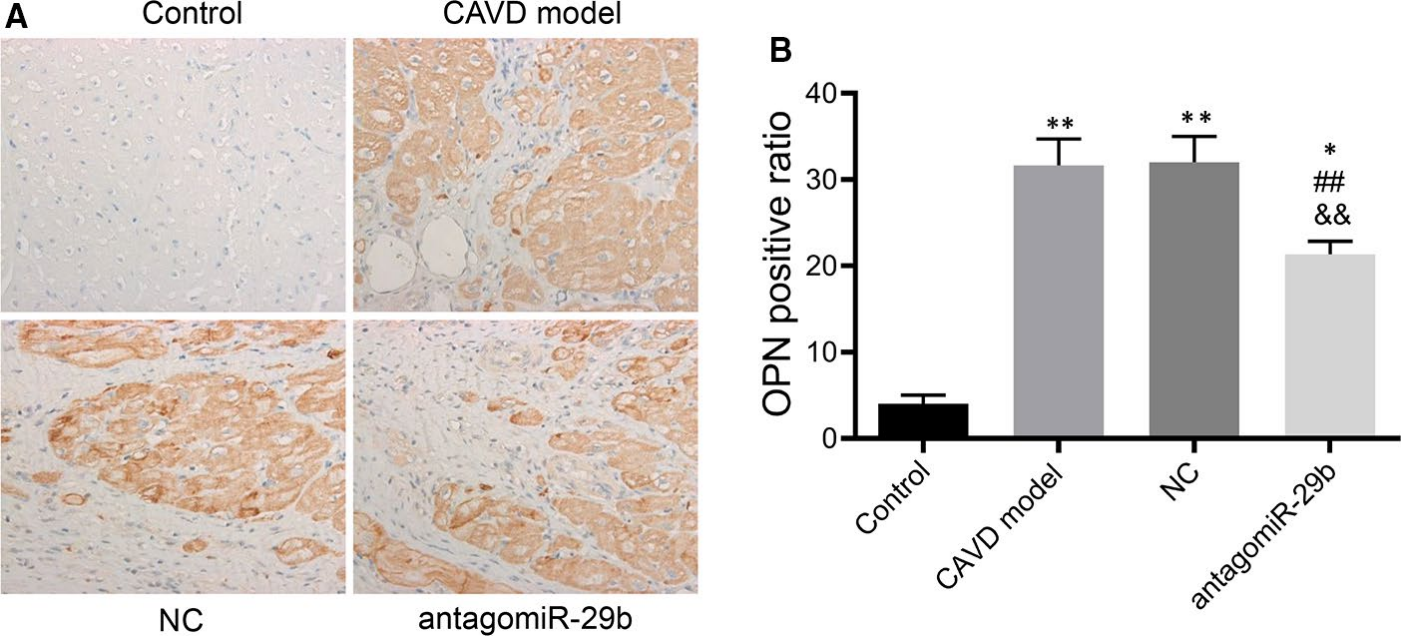
Immunohistochemistry analysis of OPN expression in the aortas of rats in each group. A, Representative images of OPN‐stained sections. B, quantitative analysis of OPN‐stained sections by ImageJ. The photomicrograph was taken at 100× magnification. The experiments were performed at least 3 times, and data are expressed as means ± SD. *P < .05, **P < .01 vs control group, ##*P* < .01 vs CAVD model group, &&*P* < .01 vs NC model group

To get insights into the potential molecular mechanism of miR‐29b in CAVD, we preformed gene expression and protein expression analyses using the aorta samples. The qRT‐PCR results indicated that the mRNA levels of osteogenic markers such as OCN, OPN, ALP and Runx2 were significantly up‐regulated in the CAVD model compared to the control group (*P* < .01, Figure 2). In addition, the same trends were observed for Wnt3 and β‐catenin mRNA levels (*P* < .01, Figure 2). However, upon antagomiR‐29b treatment, the mRNA levels of these genes were significantly decreased compared to the CAVD model group (*P* < .01, Figure 2). Meanwhile, the mRNA level of TGF‐β3 was significantly decreased in CAVD model compared to the control group, but markedly increased following the antagomiR‐29b treatment comparatively to the CAVD model group (*P* < .01, Figure 2). Besides, there was no significant difference in Smad3 expression among these four groups (*P* > .05, Figure 2). As well, Western blot results indicated that OCN, OPN, ALP, Runx2, Wnt3 and β‐catenin were up‐regulated in the CAVD model (*P* < .01, Figure 3) although declined protein expression of TGF‐β3 was observed. Similar to the mRNA expression data, the protein expression levels of OCN, ALP, Runx2, Wnt3 and β‐catenin were down‐regulated whereas the expression of TGF‐β3 was significantly promoted by antagomiR‐29b administration compared to CAVD model group (Figure 3). Additionally, although the expression of Smad3 did not differ significantly among the groups, the expression of p‐Smad3 changed under different conditions, which significantly up‐regulated in CAVD model group (*P* < .05) and NC group (*P* < .01), but significantly down‐regulated in antagomiR‐29b treatment when compared with the CAVD model (*P* < .05, Figure 3). Furthermore, immunohistochemical analysis confirmed that OPN was significantly increased in the aortas of CAVD rats but significantly decreased by antagomiR‐29b (Figure 4). The staining intensity in the cytoplasmic was the highest. When the higher power was used for observation, it was found that a large number of visible extracellular pigmented patches were existed in the neointima and media at the part or edge of the cell, or along the elasticlamellae (Figure 4). The staining intensity observed in the cultured cells was significantly lower than the neointima. However, the levels of osteopontin protein of several clusters of smooth muscle cell in the lateral medial area were almost the same as those observed in the neointima (Figure 4). After counterstaining with haematoxylin, it was showed that the labelled cells were not displayed in the adventitial layer, but were visible in the external elastic lamina.

**FIGURE 5:**
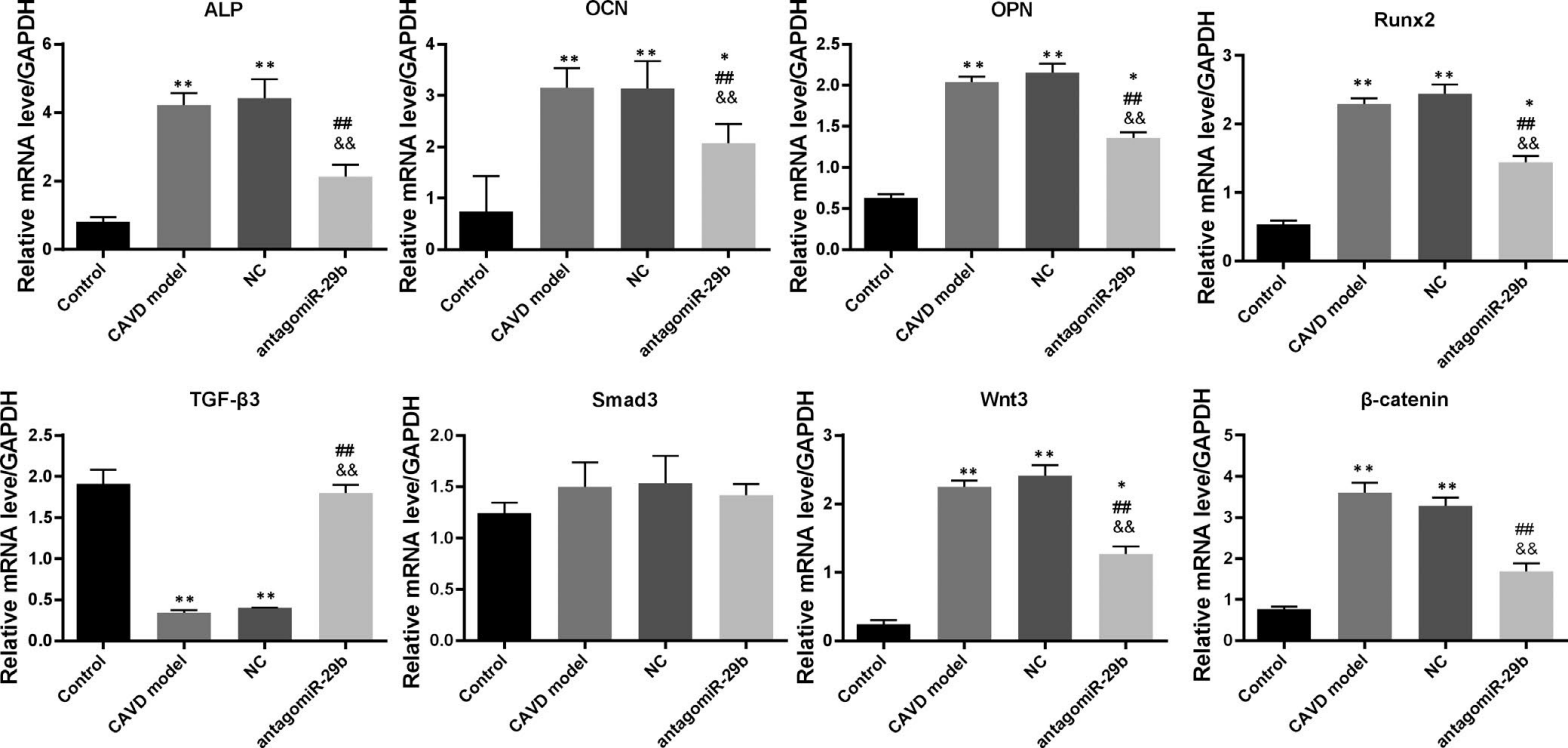
AntagomiR‐29b promoted TGF‐β3 expression and inhibited Wnt3/β‐catenin/Smad3 axis and osteogenic factors in the aortic valves of CAVD model at protein level. The mRNA expression levels of different genes were determined by qRT‐PCR. The experiments were performed at least 3 times, and data are expressed as means ± SD. *P < .05, **P < .01 vs control group, ##*P* < .01 vs CAVD model group, &&*P* < .01 vs NC model group

**FIGURE 6:**
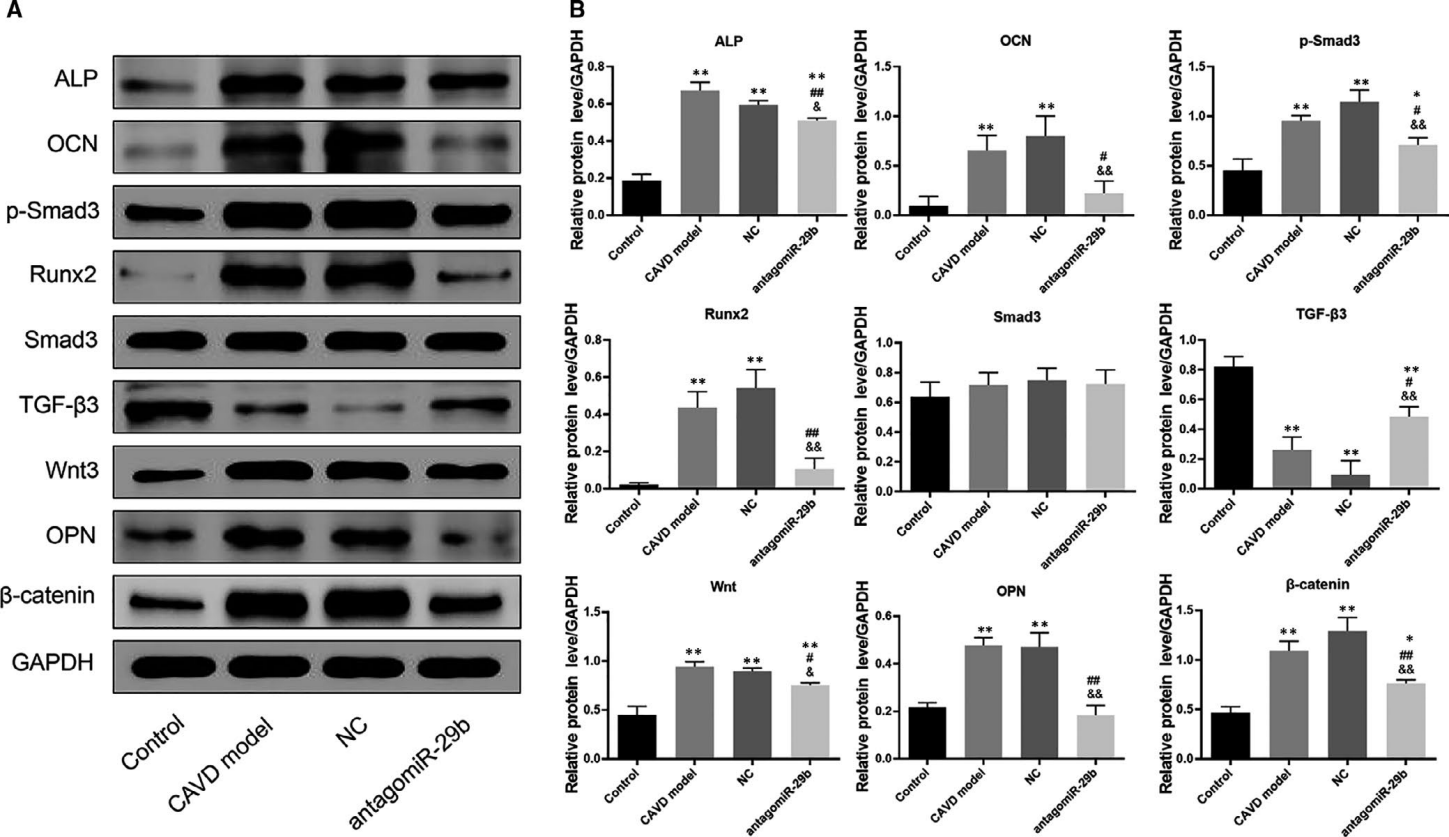
AntagomiR‐29b promoted TGF‐β3 expression and inhibited Wnt3/β‐catenin/Smad3 axis and osteogenic factors in the aortic valves of CAVD model at protein level. The protein expression levels of different genes were determined by Western blotting. The experiments were performed at least 3 times, and data are expressed as means ± SD. *P < .05, **P < .01 vs control group, #*P* < .05, ##*P* < .01 vs CAVD model group, &*P* < .05, &&*P* < .01 vs NC model group

Furthermore, qRT‐PCR was used to explore the mRNA levels of osteogenic markers in the aortic valves. The results indicated that the mRNA levels of OCN, OPN, ALP, Runx2, p‐Smad3 Wnt3 and β‐catenin were markedly increased in the CAVD model compared to the control group (*P* < .01, Figure 5). Interestingly, treatment with antagomiR‐29b significantly down‐regulated the expression of these genes compared to the CAVD model group (Figure 5). In addition, we found that TGF‐β3 mRNA level was significantly down‐regulated in the CAVD model relatively to the control group, and this trend was reversed by antagomiR‐29b administration compared to the CAVD model group (Figure 5). Similar results were observed with Western blotting experiments (Figure 6).

These results suggested that antagomiR‐29b can effectively prevent the progression of CAVD by possibly promoting TGF‐β3 through repressing the Wnt3/β‐catenin/Smad3 axis, which may serve as a potential target for treatment of CAVD.

### 3.4 AntagomiR‐29b antagonizes angiogenesis in the aorta of CAVD rat model

**FIGURE 7:**
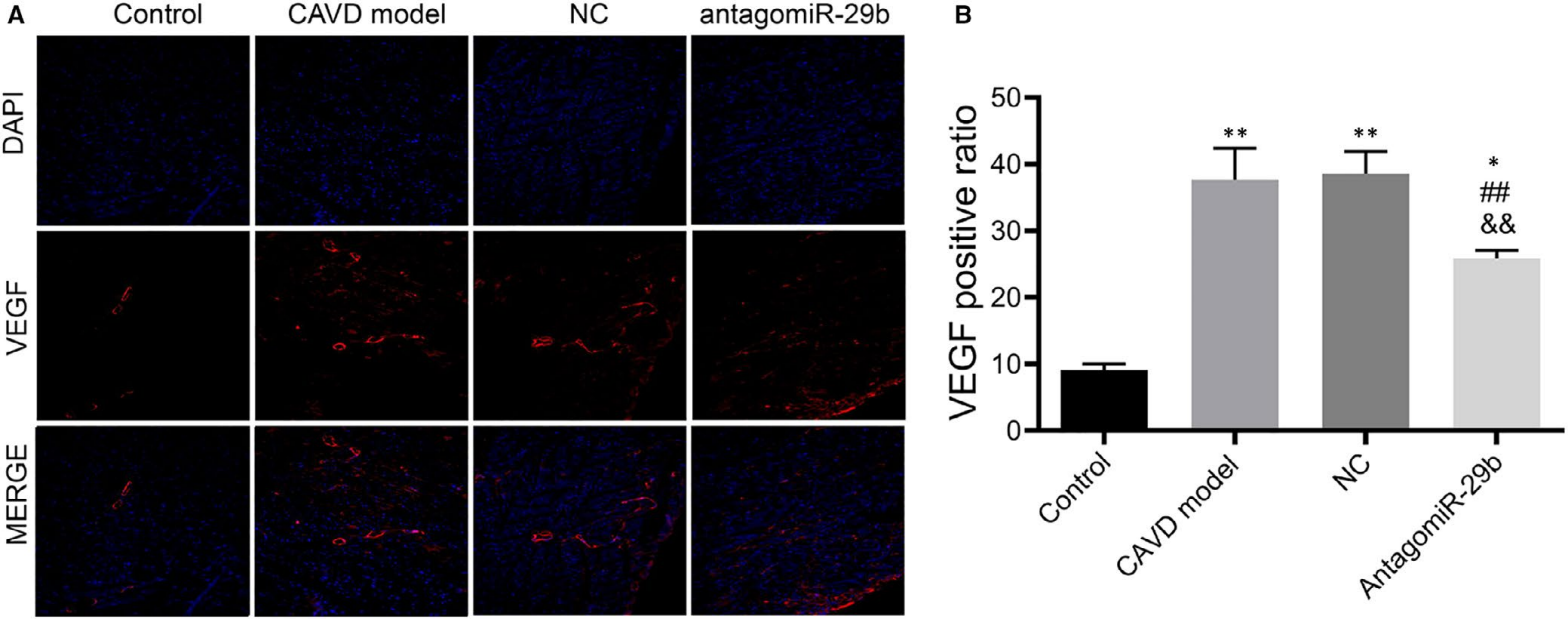
Immunofluorescence analysis of VEGF expression in the aortas of rats in each group. A, Representative images of VEGF‐stained sections. B, quantitative analysis of VEGF‐stained sections by Image J. The photomicrograph was taken at 100× magnification. The experiments were performed at least 3 times, and data are expressed as means ± SD. *P < .05, **P < .01 vs control group, ##*P* < .01 vs CAVD model group, &&*P* < .01 vs NC model group

In order to uncover whether miR‐29b impacts on the angiogenesis of the aortas of CAVD rat model, we performed VEGF immunofluorescence in aortas collected from animals in the four groups. As shown in Figure 7, we found that the expression of VEGF was significantly increased in the CAVD model group compared to the control group. In addition, antagomiR‐29b treatment significantly decreased the expression of VEGF in the CAVD rats comparatively to the CAVD model groups (Figure 7). No significant difference was observed between the CAVD model group and NC group (Figure 7). These results indicated that antagomiR‐29b antagonizes angiogenesis in the aorta of CAVD rat model.

### 3.5 AntagomiR‐29b improves heart function in CAVD rat model

**FIGURE 8:**
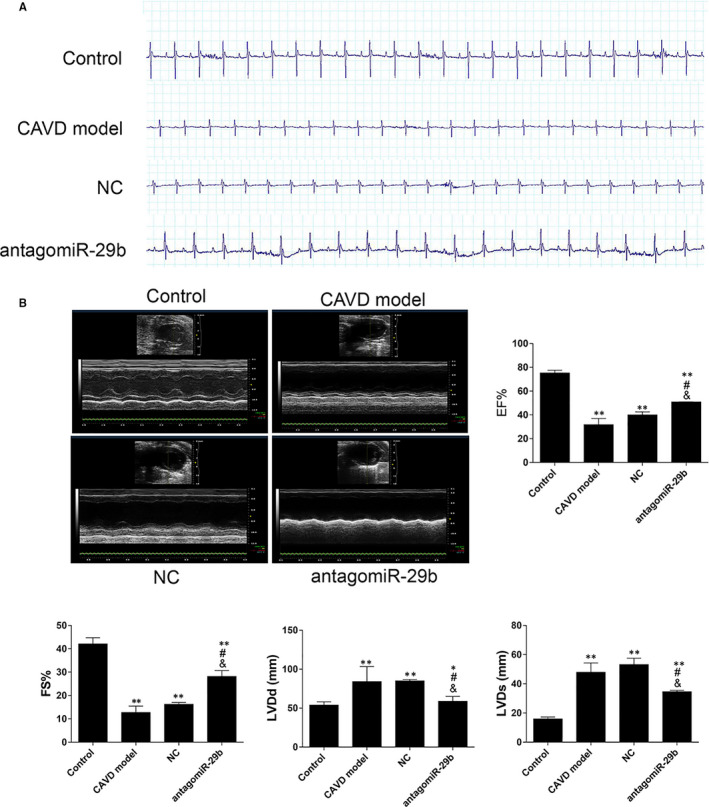
AntagomiR‐29b improves heart function in CAVD rat model. A, Representative images of electrocardiograms in different groups. B, Representative echocardiogram images and quantitative analysis of heart function parameters. The experiments were performed at least 3 times, and data are expressed as means ± SD. **P < .01 vs control group, #*P* < .05 vs CAVD model group, &*P* < .05 vs NC model group

To evaluate heart performance in CAVD model and the possible effect of antagomiR‐29b, ECG and echocardiography analyses we performed. ECG data indicated that, compared with the control group, the heart rate was significantly decreased in the CAVD model (Figure 8A). Interestingly, we found that antagomiR‐29b treatment improved the heart rate of the CAVD model (Figure 8A). Echocardiography analysis was performed to examine the cardiac performance in rats from the four groups. The M‐mode echocardiograms were depicted in Figure 8B. The results indicated that the pattern of mitral Doppler flow spectra was consistent and smooth in the control group, whereas the middle space among inferior and superior flow spectra was regular and stable (Figure 8B). However, significant variations in the middle space distance and sawtooth waves were observed in the CAVD model group, which was reverted after treatment with antagomiR‐29b group (Figure 8B). The indices of EF% and FS% were significantly decreased in the CAVD model compared to the control group but, interestingly, EF% and FS% were significantly increased upon treatment of CAVD rats by the antagomiR‐29b (Figure 8B). On the contrary, LVDd and LVDs were significantly increased in the CAVD model compared to the control (*P* < .01, Figure 8B), but decreased in the antagomiR‐29b treatment group (*P* < .01, Figure 8B). These results indicated that antagomiR‐29b improves heart function in CAVD rat model.

## 4 DISCUSSION

Previous researches have shown that vascular calcification may be related to a number of mechanisms such as calcium metabolism disorder and osteoblast phenotypic transformation of VSMCs,^15^, ^19^ but the exact mechanisms so far remain unclear. Our in vitro study showed that miR‐29b is a positive regulator of hAVICs calcification.^15^ However, there was no in vivo study conveying the potential involvement of miR‐29b in aortic calcification and its impact on heart function. Here, we uncovered that miR‐29b was increased in the cardiovascular system of CAVD rat model and showed that the inhibition of miR‐29b mitigated aortic calcification in tissues of CAVD rats. We also found that the expression of the endogenous TGF‐β3 was down‐regulated in the calcification model in vivo. Meanwhile, antagomiR‐29b treatment significantly increased the expression level of TGF‐β3 and inhibited vascular and valvular calcification, showing a decrease in calcium content and ALP activity, indicating that the endogenous miR‐29b/TGF‐β3 pathway may be involved in the processes of vascular and valvular calcification. This finding corroborated with our previous study that miR‐29b promoted the calcification of hAVICs via direct targeting of TGF‐β3.^15^

It is well known that osteoblast transformation of VSMCs is an important process of calcification.^15^, ^20^ Recent studies have shown that during calcification, the expression of smooth muscle lineage markers in VSMCs is reduced whereas the expression of osteogenic markers is increased.^21^, ^22^, ^23^ In this study, we found that the expression levels of osteogenic markers were increased in CAVD model in vivo. Nonetheless, antagomiR‐29b treatment significantly reverted the expression of these osteogenic markers. These results suggested that antagomiR‐29b inhibits aortic and valvular osteoblastogenesis. These findings corroborated with our previous in vitro studies.^15^ Other studies have also indicated that miR‐29b is intrinsically involved in osteogenesis.^24^, ^25^, ^26^

Moreover, we further investigated the potential mechanism of inhibiting CAVD through antagomiR‐29b. TGF‐β is known to regulate cell differentiation, proliferation and apoptosis through cell surface receptor signal transduction pathway.^27^, ^28^ Previous studies have shown that TGF‐β1 regulates interstitial cell calcification through apoptosis mechanism in calcified aortic valves.^29^ Moreover, TGF‐β can also regulate vascular calcification and the differentiation of VSMCs.^30^ TGF‐β transmits cytoplasmic signals into the intracellular domain through phosphorylating Smad2 and Smad3 by activating type II receptor.^31^ The activated phosphorylated Smad2 and Smad3 regulate the transcription of a series of genes via binding to Smad4.^32^ In our study, we found that the expression of TGF‐β3 was decreased, whereas its downstream signal molecule Smad3 was significantly increased in the CAVD model in vivo. We also found that treatment with antagomiR‐29b significantly promoted the expression of TGF‐β3 and the expression of Smad3 was inhibited. Previous researches have shown that Runx2 is an important regulator of vascular calcification.^33^ Up‐regulation of Runx2 is crucial for the calcification process, which was observed in vascular calcification of chronic kidney disease patients.^34^ In addition, Runx2 acts as a significant target gene for the TGF‐β signal pathway, can inhibit myogenic differentiation of C2C12 cells and induce osteoblast differentiation.^35^ Here, we found up‐regulation of Runx2 expression in the CAVD model in vivo, although antagomiR‐29b declined the expression of Runx2. These results demonstrated that the miR‐29b/TGF‐β3 axis may be possibly involved in the mechanisms underlying CAVD. In addition, the expression of OPN, which is closely related to bone formation, was markedly increased in CAVD aortas compared to the control group, which was significantly corroborated with elevated expression in the valve tissue of vascular calcification patients.^36^

Our study showed that the expression of VEGF in the CAVD model was significantly increased compared to the control group but reverted by antagomiR‐29b administration, which was corroborated by its role in the occurrence and development of vascular calcification.^16^ As VEGF is an angiogenic marker, we also stipulated that angiogenesis occurs in CAVD and that antagomiR‐29b exerts anti‐angiogenic effect in CAVD. This hypothesis was supported by the previous findings indicating that miR‐29b hinders angiogenesis in hepatocellular carcinoma.

Moreover, we found that CAVD negatively impacts on heart performance and that this effect is suppressed by the miR‐29b inhibition. This indicated that miR‐29b may be a key therapeutic target in CAVD and cardiovascular diseases such as vascular stenosis and heart failure.

Nevertheless, this study has some limitations. Studies have shown that vascular calcification is associated with decreased vascular compliance and arterial hypertension.^37^ Vitamin D3 plus nicotine can cause severe calcification in the rat aorta and significantly increase blood pressure.^38^ Our current study indicates that antagomiR‐29b treatment can significantly attenuate vascular and valvular calcification in rats, but does not involve blood pressure studies. Therefore, we cannot rule out the possibility that blood pressure may also be related to the inhibition of vascular calcification by miR‐29b and these problems need to be further clarified.

In summary, this study provides important new insights into the mechanisms of miR‐29b in CAVD and opens the door for innovative preventative strategies by providing new targets for small molecule therapies. Specifically, pharmacological inhibitors that can prevent miR‐29b up‐regulation during early development may represent an intriguing therapeutic approach.

## CONFLICT OF INTEREST

The authors declare that they have no conflicts of interest with the contents of this article.

## AUTHOR CONTRIBUTIONS

**Ming Fang:** Conceptualization (equal); Data curation (equal); Formal analysis (equal); Funding acquisition (equal); Validation (equal); Writing‐original draft (equal); Writing‐review & editing (equal). **Kangyong Liu:** Data curation (equal); Formal analysis (equal); Funding acquisition (equal); Investigation (equal); Validation (equal); Writing‐original draft (equal); Writing‐review & editing (equal). **Xinming Li:** Funding acquisition (equal); Investigation (supporting); Methodology (supporting); Software (supporting). **Yudai Wang:** Investigation (supporting); Resources (supporting); Software (supporting). **Wei Li:** Conceptualization (lead); Supervision (lead); Validation (lead). **Bin Li:** Conceptualization (supporting); Formal analysis (lead); Project administration (lead); Supervision (lead); Validation (lead); Writing‐review & editing (lead).

## Data Availability

All data generated or analysed were published within the manuscript.
